# Oteseconazole *versus* fluconazole in the treatment of acute candidiasis: a systematic review and meta-analysis

**DOI:** 10.61622/rbgo/2026rbgo32

**Published:** 2026-07-17

**Authors:** Elizabeth Honorato de Farias, Ana Gabriela Alves Pereira, Anderson Matheus Pereira da Silva, Lucas Silva Cabeça, Laila Leite Pacheco Vieira, Pedro Lucas Machado Magalhães, Josvaldo da Silva Viana, José Eleutério

**Affiliations:** 1 Universidade Federal de Roraima Boa Vista RR Brazil Universidade Federal de Roraima, Boa Vista, RR, Brazil; 2 Universidade Estadual Paulista Botucatu SP Brazil Universidade Estadual Paulista, Botucatu, SP, Brazil; 3 Universidade Federal de Pernambuco Recife PE Brazil Universidade Federal de Pernambuco, Recife, PE, Brazil; 4 Universidade Federal do Pará Belém PA Brazil Universidade Federal do Pará, Belém, PA, Brazil; 5 Centro Universitário CESMAC Maceió AL Brazil Centro Universitário CESMAC, Maceió, AL, Brazil; 6 Instituto de Educação Médica Angra dos Reis RJ Brazil Instituto de Educação Médica, Angra dos Reis, RJ, Brazil; 7 Universidade Federal do Ceará Fortaleza CE Brazil Universidade Federal do Ceará, Fortaleza, CE, Brazil

**Keywords:** Vulvovaginal candidiasis, Fluconazole, Oteseconazole

## Abstract

**Objective:**

Vulvovaginal candidiasis (VVC) is a common infection traditionally treated with fluconazole. Oteseconazole is a new oral antifungal offering a promising alternative. However, it is essential to assess if it has better efficacy than the treatment already used in VVC.

**Methods:**

This systematic review and meta-analysis followed PRISMA guidelines, searching PubMed, EMBASE, and Cochrane for studies comparing oteseconazole and fluconazole in acute VVC. Three randomized controlled trials were included. Odds ratios were calculated using a random-effects model.

**Results:**

The initial search yielded 203 results, but only three RCTs with 593 patients were included. Oteseconazole showed significantly higher rates of therapeutic and sustained clinical cure at day 14 and 28 compared to fluconazole (OR 2.56 and 2.08, respectively), with similar rates of mild to moderate adverse events. Risk of bias was high due to selective reporting.

**Conclusion:**

Oteseconazole appears to be a safe and more effective alternative to fluconazole for acute VVC, but further research and more robust studies are needed to confirm these findings.

**PROSPERO Registry**: #CRD420251010309

## Introduction

Vulvovaginal candidiasis is a fungal symptomatic infection of the genital mucosa characterized by yeast colonization, with *Candida albicans* being the most frequent etiological agent. However, diseases caused by other *Candida* sp., particularly *Candida glabrata,* have increasingly been recognized as relevant in VVC.^(1,2)^

Although *Candida* sp. is part of the vaginal microbiome, factors such as stress, immunosuppression, and the use of certain medications can disrupt this microbial balance and *Candida* overgrowth. Besides, this imbalance can enable the fungus to transition from a commensal to a pathogenic state, leading to the development of the symptoms.^(2,3)^

It is estimated that approximately 80% of women will experience at least one episode of VVC during their lifetime, with a substantially higher incidence among women of reproductive age compared to those in postmenopause. Additionally, approximately 50% of these individuals are affected by recurrent episodes, known as recurrent vulvovaginal candidiasis (RVVC).^(2,4)^

The clinical findings of this infection include: erythema; vulvar fissures; pruritus; discharge; vulvar edema; excoriations; introital dyspareunia, and dysuria.^(5)^ These manifestations can be assessed using the Vulvovaginal Signs and Symptoms Score (VSS), a standardized scoring system that classifies signs and symptoms according to severity. Each clinical sign (congestion and edema, excoriations, fissures, and erosions, in addition to the amount of dysfunction) and symptom reported by the patient (pruritus and pain) receives a score from 0 to 3 (0= absent, 1= mild, 2= moderate and 3= severe), except for the item "excoriations, fissures and erosions", which is scored as 0 when absent and 3 when presente.^(2)^

Given the recurrent nature of this infection and the severity of its symptoms, VVC typically affects women's quality of life, leading to higher levels of stress and decreased self-esteem and self-confidence.^(6)^ Thus, the appropriate management of VVC remains an ongoing challenge for patients and gynecologists, requiring effective therapeutic strategies and a careful approach to disease control.^(2)^

According to the Infectious Diseases Society of America Guidelines (IDSA) and The American College of Obstetricians and Gynecologists (ACOG) for the treatment of RVVC, the first-line treatment is fluconazole, administered orally for 10 to 14 days, followed by a maintenance regimen of 150 mg of fluconazole once a week for six months.^(1,7,8)^ However, up to 50% of women experience symptom recurrence within six months after discontinuing treatment, necessitating additional rounds of induction and maintenance therapy.^(8,9)^

The options for antifungal treatments are alarmingly limited, and insufficient research has been dedicated to understanding which treatments are the most effective. This knowledge gap highlights the need for a deeper exploration of our therapeutic choices in combating fungal infections. Given this scenario, the search for alternative, effective therapeutic options is crucial for managing this infection appropriately.^(10)^ Therefore, we conducted the first independent meta-analysis to estimate the efficacy and safety oteseconazole compared to fluconazole in acute VVC.

## Methods

This systematic review and meta-analysis were based on the Cochrane Handbook and the Preferred Reporting Items for Systematic Review and Meta-Analysis (PRISMA) statement.^(11,12)^ The protocol was registered in the International Prospective Register of Systematic Reviews (PROSPERO) registration number CRD420251010309.

The review question was: *"How does the efficacy of oteseconazole compare to fluconazole in treating acute vulvovaginal candidiasis in women?".* The questions were formulated using the PICO(TT) framework, with the following elements: (P): Women with acute candidiasis; (I): Use of oteseconazole; (C): Use of fluconazole; (O): Mycological cure, adverse events; (T): Therapeutic, randomized; (T): No restrictions. Studies published up to January 2025 were eligible, and no language restrictions were applied.

Studies were included if they met the following criteria: (1) enrolled adult women diagnosed with acute vulvovaginal candidiasis, (2) compared the use of oteseconazole and fluconazole as antifungal treatments, (3) reported outcomes related to treatment efficacy, recurrence rate, or adverse effects, and (4) were designed as randomized controlled trials (RCTs).

Studies were excluded in cases of: (1) observational studies, case reports, review articles, letters, editorials, conference abstracts, or systematic reviews; (2) including populations other than adult women with acute vulvovaginal candidiasis; (3) did not involving a direct comparison between oteseconazole and fluconazole; or (4) did not report relevant clinical outcomes or presented insufficient or unclear data.

The research presented in this systematic review and meta-analysis followed PRISMA guidelines.^(11)^ The authors conducted an extensive search of PubMed, Embase, and the Cochrane Central Register of Controlled Trials from inception to December 2024. Boolean operators ("AND" and "OR") were applied to combine the following search terms: "Vulvovaginal Candidiasis", "Candida albicans" combined with treatment-related terms such as "fluconazole" and investigational agents such as "oteseconazole". The search strategy was developed using the PRESS (Peer Review of Electronic Search Strategies) method to ensure rigor and comprehensiveness. The complete electronic search strategies for all databases are provided in Supplementary Material Table 1S and 2S.

All retrieved records were uploaded to the Rayyan platform (https://www.rayyan.ai/) to facilitate the screening process. During the initial phase, titles and abstracts were independently reviewed by two authors (L.L and A.G), and any disagreements were resolved by a third author (E.H). Studies that did not directly address the research question or were duplicates were excluded. In the second phase, full-text articles were assessed for eligibility based on the predefined inclusion and exclusion criteria. Any disagreements were resolved through discussion. Additionally, the reference lists of included studies and relevant systematic reviews were manually screened to identify any potentially eligible articles not captured in the initial search. Searches were also extended to include conference abstracts and prospective clinical trials to ensure the comprehensiveness of the evidence base.

Data extraction was conducted by a single author (L.S), who gathered key information from each study, including publication year, study design, sample size, patient age, and relevant outcomes. The extracted data were then reviewed by a second author (E.H). For studies with missing summary statistics, we employed imputation methods following Cochrane guidelines and standardized any non-uniform units. Results were organized and visually displayed using spreadsheets to support systematic synthesis and comparison across studies.

*The primary outcomes of this study were: (1) treatment response, (2) sustained clinical response, and (3) safety and tolerability of oteseconazole.*

Treatment response was defined as the clinical resolution of signs and symptoms of acute vulvovaginal candidiasis following the initial treatment. Sustained clinical response was defined as the maintenance of symptom resolution without recurrence over the follow-up period defined by each included study. Safety and tolerability were evaluated based on the incidence of adverse events, which were categorized into neurologic, infection-related, and gastrointestinal events, as reported by the original studies. All outcomes were assessed as dichotomous variables and analyzed using odds ratios with 95% confidence intervals.

The Risk of Bias 2 (ROB2) tool was used to assess the risk of bias and quality assessment in randomized studies. Two independent authors (P.L and J.S) completed the risk of bias assessment. After discussing the reasons for the discrepancy, disagreements were resolved through consensus. Additionally, we didn't perform a funnel plot test to evaluate reporting biases because no endpoint had at least ten included studies in their respective meta-analysis. Disagreements in the quality assessment were resolved through mutual consensus.

We conducted the meta-analyses using Odds Ratio (OR) with 95% Confidence Intervals (CIs) for binary outcomes. The Mantel-Haenszel method with a random-effects model was applied to account for potential heterogeneity among studies. Statistical heterogeneity was assessed using the Cochran's Q test and quantified by the I^2^ statistic; values of I^2^>40% and p-values<0.10 indicated significant heterogeneity. For outcomes with substantial heterogeneity (I^2^≥ 40%), a leave-one-out sensitivity analysis was performed to assess the robustness of the pooled estimates and to identify studies with a high influence on overall heterogeneity. All statistical analyses were conducted using R software (version 4.4.0; R Foundation for Statistical Computing, Vienna, Austria).

## Results

**Figure 1:**
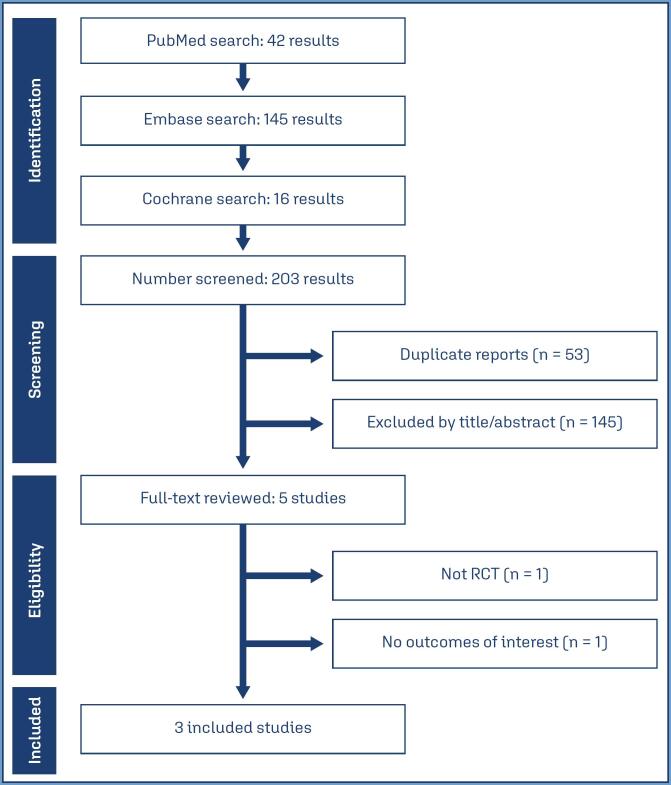
The figure is available in color online only PRISMA flow diagram of study screening and selection according to the PRISMA template

The initial search yielded 203 results from the following five databases:42 results from PubMed, 145 from Embase, and 16 from Cochrane. After removing 53 duplicates, 150 articles underwent initial screening, from which 145 were excluded based on the information provided in the abstract and title, leaving five articles for full-text assessment for eligibility. Ultimately, three RCTs met our inclusion criteria. The PRISMA Flow Diagram is shown in figure 1, detailing the reasons for excluding the articles that underwent full-text assessment.

A total of 593 patients from three RCTs, Wang et al.,^(2)^ UltraVIOLET (Martens et al.,^(8)^ and Brand et al.,^(13)^ were included in this systematic review and meta-analysis. Oteseconazole was prescribed to 347 (58.22%) patients, and Fluconazole to 246 (41.78%). Study characteristics are detailed in chart 1. The mean follow-up ranged from 1 to 9 months, with the overall mean age being 32.7 years in Brand's study, 30.55 years in Wang's study, and 35.0 years (SD 11.0) in the UltraVIOLET study. Brand's study included healthy, nonpregnant women aged ≥18 and <65 with symptomatic VVC, while Wang's study enrolled women aged ≥18 and ≤75 with a vulvovaginal signs and symptoms score ≥7. The UltraVIOLET study included women with a history of RVVC (≥3 episodes in the past 12 months) and a confirmed acute VVC episode at screening, defined by a composite signs and symptoms score ≥3 (erythema, edema, excoriation, itching, burning, and/or irritation). *C. albicans* was the predominant species across studies, with 41 isolates in Brand's, 249 in Wang's, and 88 (40%) in UltraVIOLET. The data is summarized in chart 1.

**Chart 1:** Baseline characteristics of included studies

Studies			Population					Candida species		
Reference	Design	Country	Patients OTZ/FCZ (n)	Follow-up (m)	Age † (y)	BMI † (kg/m^2^)	Inclusion criteria	C. albicans (n)	C. glabrata (n)	C. parapsilosis (n)
Brand et al. (2021)^(13)^	RCT	USA	40/15	6	32.7 ± 10.40	27.9 ± 6.27	Healthy, nonpregnant female participants aged ≥18 years and <65 years with a clinical diagnosis of symptomatic acute VVC were enrolled in the study	41	1	9
Martens et al. (2022)^(8)^	RCT	USA	147/72	9	35(11.0)	29(8)	History of RVVC, defined as 3 episodes of acute VVC in the past 12 months; confirmation of acute VVC episode at screening, defined as a total score of 3 for vulvovaginal signs (erythema, edema, and/or excoriation) and symptoms (itching, burning, and/or irritation).	88(40)	4(2)	1(<1)
Wang et al. (2024)^(2)^	RCT	China	160/159	1	29.9 (8.0) / 31.2 (7.5)	21.4 (3.5) / 21.5 (3.2)	Female subjects aged ≥18 and ≤75 years with vulvovaginal signs and symptoms (VSS) score ≥7 were enrolled	249	49	3

† mean (SD) or median (range) or n (%);

Kg/m^2^: Kilograms per square meter; m: months; n: number; RCT: Randomized Controlled Trial; USA: United States of America; y: year; NR: not reported

**Figura 2:**
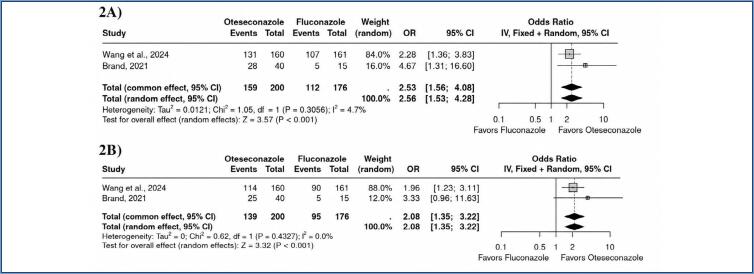
Forest plot showing the odds ratio for clinical response comparing Oteseconazole and Fluconazole. (A) D14 days (B) D28 days

In a meta-analysis of three studies, data on treatment response were available from two studies, which were included in the forest plot. The treatment response rate was higher in the oteseconazole group compared to fluconazole (OR 2.53; 95% CI 1.56–4.08) (Figure 2A). In the random-effects model, the pooled estimate favored oteseconazole (OR 2.56; 95% CI 1.53–4.28; I^2^ = 4.7%) (Figure 2A). Although three studies were included in the meta-analysis, only two provided data on sustained clinical response at 28 days and were therefore included in the forest plot. The pooled analysis showed a higher sustained response rate in the oteseconazole group than in the fluconazole group (OR 2.08; 95% CI 1.35–3.22; I^2^ = 0.0%) (Figure 2B).

**Figure 3:**
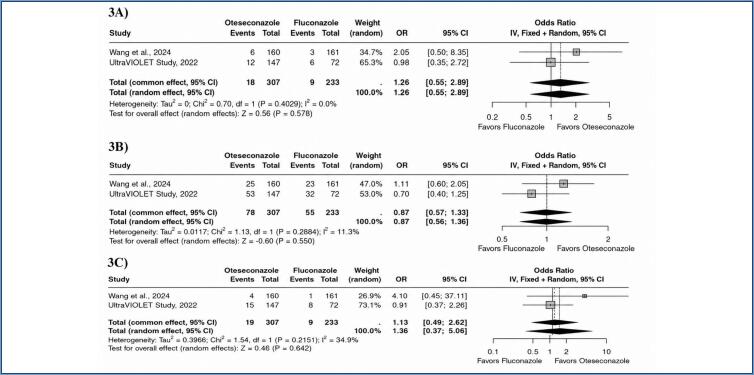
Forest plot showing the adverse events incidence. 3A) Neurologic Disorders; 3B) Infections and Infestations; 3C) GI Disorders

The incidence of neurologic adverse events (headache, dizziness, nausea, and lethargy) was slightly higher, but not significant in the oteseconazole group compared to fluconazole (OR 1.26; 95% CI 0.55–2.89; I^2^ = 0.0%; p = 0.578) (Figure 3A). Infection-related adverse events were comparable between groups (OR 0.87; 95% CI 0.56–1.36; I^2^ = 1.3%; p = 0.550) (Figure 3B). Gastrointestinal adverse events were not significantly different between the groups (OR 1.36; 95% CI 0.37–5.06; I^2^ = 34.9%; p = 0.642) (Figure 3C).

**Figure 4:**
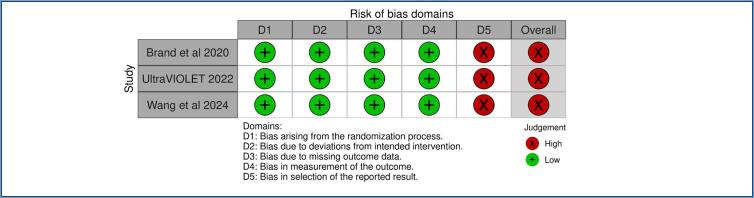
Risk of bias summary for randomized studies (RoB 2)

The included studies were assessed with the RoB 2 tool, covering five domains. All three studies had adequate randomization (Domain 1), proper blinding (Domain 2), minimal and balanced follow-up losses (Domain 3), and standardized, blinded outcome measurement (Domain 4), resulting in low risk of bias for these domains. However, all studies showed a high risk of bias in Domain 5 due to multiple eligible outcome measurements and analyses without clear pre-specification, raising concerns about selective reporting. Consequently, the overall risk of bias was rated as high for all studies because of this issue with outcome reporting (Figure 4).

The certainty of evidence was evaluated using the GRADE approach. For treatment response, certainty was moderate due to serious risk of bias from selective reporting and inconsistency in outcome timing (14–28 days). Despite these issues, a strong association supported the findings. Sustained clinical response had high certainty, with no major concerns and a strong intervention-outcome link. Certainty for neurological adverse events was low, reflecting serious risk of bias and inconsistency from varied definitions. For infection-related and gastrointestinal adverse events, certainty was moderate, mainly due to risk of bias, with no concerns about inconsistency, imprecision, or indirectness. Full justifications are detailed in the Supplementary Material Table 3S.

## Discussion

This systematic review and meta-analysis, which included three RCTs with 593 patients, evaluated the efficacy of oteseconazole compared to fluconazole in treating acute vulvovaginal candidiasis. Across randomized controlled trials, oteseconazole demonstrated a higher rate of therapeutic cure at day 28, with one large phase 3 trial reporting significantly greater clinical and mycological cure rates for oteseconazole versus fluconazole (therapeutic cure: 66.9% vs 45.9%, P = 0.0002; mycological cure: 82.5% vs 59.1%, P < 0.0001) in women with severe VVC.^(2)^ These findings are consistent with earlier phase 2 data, which showed numerically higher—but not statistically significant—therapeutic cure rates for oteseconazole, and notably lower rates of mycological recurrence at 3 and 6 months compared to fluconazole.^(13)^

Overall, oteseconazole demonstrated a better treatment response and a higher sustained clinical response (OR 2.53; 95% CI 1.56–4.08). In a random-effects model, the pooled estimate favored oteseconazole (OR 2.56; 95% CI 1.53–4.28; I^2^ = 4.7%).

Sustained clinical response at 28 days was significantly higher in the oteseconazole group (OR 2.08; 95% CI 1.35–3.22; I^2^ = 0.0%). However, the results were inconsistent: while one RCT showed significantly better outcomes with oteseconazole, the other found no meaningful difference between the two treatments. Concerning adverse events, although oteseconazole showed a slightly higher incidence of neurological effects (OR 1.26; 95% CI: 0.55–2.89) and gastrointestinal effects (OR 1.36; 95% CI: 0.37–5.06), the differences were not statistically significant, and the wide confidence intervals for these safety outcomes, stemming from a low number of events, prevent firm conclusions regarding the comparative safety profile. Neurological disorders (e.g., headache, dizziness) and gastrointestinal disturbances such as nausea, diarrhea, and abdominal discomfort were slightly more frequent compared to fluconazole. A slight but notable increase in infection-related adverse events, including upper respiratory tract infections, was also observed.

The vaginal microbiota plays a fundamental role in maintaining vaginal health, primarily through the dominance of *Lactobacillus* spp. — including *L. crispatus*, *L. gasseri*, and *L. jensenii*. These species help preserve a low vaginal pH and inhibit the growth of opportunistic pathogens. In VVC, although *Lactobacillus* levels may decrease, the disruption does not typically involve an overgrowth of anaerobic bacteria, as seen in bacterial vaginosis (BV), but rather an overproliferation of *Candida* species. In cases where features of both conditions coexist, mixed vaginitis may be considered.^(14,15)^

The pathophysiology of VVC involves a combination of fungal virulence factors and the host's immune response. *Candida albicans*, typically a harmless commensal organism, becomes invasive during infection by transitioning to a hyphal form. This form expresses virulence factors such as secreted aspartyl proteases and candidalysin, which promote tissue invasion. While neutrophil recruitment is an essential immune response, it may unintentionally worsen inflammation without significantly reducing the fungal burden, contributing to symptoms such as pruritus vulvae, vulvovaginal erythema, dysuria, dyspareunia, and abnormal vaginal discharge.^(16,17)^ This complex host-pathogen interaction underscores the need for antifungal agents that can effectively control fungal proliferation while minimizing inflammatory damage - a therapeutic challenge that helps explain the differential outcomes observed between fluconazole and oteseconazole in clinical trials. These pathogenic mechanisms provide a biological rationale for the observed differences in clinical efficacy between fluconazole and oteseconazole.

The apparent superior clinical outcomes observed with oteseconazole compared to fluconazole can be explained by their distinct mechanisms of action. Both drugs are azole antifungals that inhibit fungal CYP51 (lanosterol 14α-demethylase), disrupting ergosterol synthesis in the fungal cell membrane. fluconazole, a triazole antifungal, has long been the standard treatment for VVC due to its broad activity against *Candida* species and convenience in oral administration. However, fluconazole, while effective, has limitations: it is primarily fungistatic, has a shorter duration of action, and is susceptible to resistance mechanisms in some species — especially in *C. glabrata*, where resistance has become increasingly concerning, particularly with repeated or prolonged use. In contrast, oteseconazole was specifically designed with enhanced properties — it binds more selectively and tightly to fungal CYP51, has minimal interaction with human cytochrome P450 enzymes (reducing side effects), and maintains prolonged antifungal activity in tissues.^(18)^ Oteseconazole has a potent activity against a broad spectrum of *Candida* species, including fluconazole-resistant strains such as *C. glabrata*, as reflected by its substantially lower minimum inhibitory concentrations (MICs) compared to fluconazole.^(19)^

In this context, the present meta-analysis evaluated the comparative efficacy and safety of oteseconazole and fluconazole in treating acute VVC. The findings suggest that oteseconazole may offer potential advantages, particularly in cases involving antifungal resistance or risk of recurrence. The clinical implications of these findings point towards a targeted use of oteseconazole. Based on its mechanism and efficacy profile, patient subgroups most likely to benefit are those with documented or suspected fluconazole-resistant infections (e.g., involving *C. glabrata*) and those with a history of frequent recurrences who have failed conventional fluconazole therapy. However, the clinical applicability of these findings is constrained by the limited number of included studies and the modest total sample size. Furthermore, the high risk of bias identified in all three studies, particularly in Domain 5 of the ROB2 tool concerning the selection of reported results, undermines the certainty of the evidence. This bias increases the potential for selective reporting and likely contributed to the observed variability in outcomes. Future trials should pre-specify outcomes in protocols to minimize this risk.

The generalizability of the results is also affected by variability in patient populations, follow-up duration, and outcome definitions across the included studies. It remains unclear which patient subgroups (e.g., based on disease severity, causative *Candida* species, or microbiome composition) are likely to derive the most significant benefit from oteseconazole. Short follow-up periods in the available literature prevent a thorough assessment of long-term efficacy and recurrence rates. While oteseconazole appears promising, further studies with larger sample sizes, extended follow-up, and more homogeneous populations are needed to confirm its long-term efficacy, better characterize its safety profile, and guide personalized clinical decision-making.

Several limitations must be noted in this study. All three studies had a high risk of bias, particularly in Domain 5, due to multiple eligible outcome measurements and analyses without clear pre-specification, increasing the potential for selective reporting. This represents a significant limitation, as the substantial risk of bias likely influenced outcome variability, potentially confounded by patient demographics, microbiome composition, and study design. Furthermore, the risks associated with fluconazole, including hepatotoxicity, drug interactions, and teratogenicity, underscore the need for more research into alternative treatments and personalized therapy.

## Conclusion

This systematic review and meta-analysis suggest that oteseconazole may be an effective alternative to fluconazole for treating acute vulvovaginal candidiasis, demonstrating superior clinical and sustained response in the short term, particularly against fluconazole-resistant *Candida* species. However, the conclusions are limited by the small number of RCTs, a high risk of bias in the existing evidence, and unexplained heterogeneity for some outcomes. Although no significant differences were observed in safety outcomes, the available data on adverse events are too limited to draw definitive conclusions about its safety profile. These findings underscore the therapeutic potential of oteseconazole but highlight the need for more robust, long-term studies to confirm its efficacy and safety and to guide clinical decision-making.

## Data Availability

The research data are described in the article presented.
