# Supplementary material for: Oteseconazole versus fluconazole in the treatment of acute candidiasis: a systematic review and meta-analysis
Source: Rev Bras Ginecol Obstet. 2026 Jul 17;48:e-rbgo32. doi: 10.61622/rbgo/2026rbgo32 (PMC13399485; doi:10.61622/rbgo/2026rbgo32)
Supplement: Supplementary Material [file 1806-9339-rbgo-48-e-rbgo32-Suppl01.pdf]

Supplementary Material

Table 1S. Complete search strategies

**Database: PubMed**<br>**Date of search: 31 January 2025**<br>**Results: 42 records**<br>((“Vulvovaginal candidiasis” OR “Candidiasis, Vulvovaginal”[Mesh] OR “acute infections” OR candidiasis OR “Candidiasis”[Mesh]) AND(fluconazole OR “Fluconazole”[Mesh] OR diflucan OR “antifungal agent fluconazole” OR “triazole antifungal” OR “antimycotic fluconazole”) AND(oteseconazole OR VT-1161 OR vivjoa OR tavaborole OR OTZ))
**Database: Embase**<br>**Date of search: 31 January 2025**<br>**Results: 145 records**<br>(‘vulvovaginal candidiasis’/exp OR ‘vulvovaginal candidiasis’ OR ‘vaginal yeast infection’ OR ‘vaginitis, yeast’<br>OR ‘acute vulvovaginal candidiasis’ OR ‘recurrent vulvovaginal candidiasis’<br>OR candidiasis OR ‘fungal vaginitis’ OR ‘mycotic vaginitis’ OR ‘candida albicans’ OR ‘candida glabrata’ OR ‘candida tropicalis’ OR ‘candida parapsilosis’ OR ‘candida auris’ OR ‘opportunistic infections’ OR ‘mycoses’) AND<br>(fluconazole OR diflucan OR ‘azole compounds’ OR ‘triazole antifungal’ OR ‘antimycotic fluconazole’ OR itraconazole<br>OR posaconazole OR voriconazole OR ketoconazole OR isavuconazole) AND(oteseconazole OR ‘VT-1161’ OR ‘Vivjoa’ OR OTZ OR ‘novel azoles’ OR ‘sterol biosynthesis inhibitors’ OR ‘lanosterol 14-alpha-demethylase inhibitors’)
**Database: Cochrane Library**<br>**Date of search: 31 January 2025**<br>**Results: 16 records**<br>((“Vulvovaginal Candidiasis” OR vulvovaginal candidiasis OR “Vaginal Yeast Infection” OR “Vaginitis, Yeast” OR candidiasis)AND(fluconazole OR itraconazole OR azole) AND(oteseconazole OR VT-1161 OR Vivjoa))

Table 2S. PRESS Checklist

PRESS Domain	Description	Assessment
Translation of the research question	The research question was appropriately translated into searchable concepts reflecting population, intervention, and condition.	Adequate
Boolean and proximity operators	Boolean operators (AND/OR) were correctly applied to combine concepts and synonyms.	Adequate
Subject headings	Controlled vocabulary terms (e.g., MeSH, Emtree) were appropriately selected and combined with free-text terms.	Adequate
Text word searching	Relevant synonyms, spelling variations, and related terms were included to maximize sensitivity.	Adequate
Spelling, syntax, and line numbers	Spelling, syntax, parentheses, and field tags were checked and found to be accurate.	Checked
Limits and filters	No limits or filters (e.g., language, publication date) were applied to the searches.	Not applied
Overall structure and completeness	The overall search strategy was comprehensive, transparent, and reproducible across databases.	Adequate

Table 3S. GRADE (Grading of Recommendations Assessment, Development and Evaluation)

Certain assessment							Summary of findings					
							Nº of patients		Effect			
Nº of studies	Study design	Risk of bias	Inconsistency	Indirectness	Imprecision	Other considerations	Oteseconazole	Fluconazole	Relative (95% CI)	Absolute (95% CI)	Certain	Importance
Treatment response (assessed with: Odds Ratio)												
2	RCT	Serious[a]	Serious[b]	Not serious	Not serious	Strong association	159/200 (79.5%)	112/176 (63.6%)	OR 2.53 (1.56-4.08)	179 more/1.000 (96-241; more-more)	Moderate	Critical
Sustained clinical response (assessed with: Odds Ratio)												
2	RCT	Serious[a]	Not serious	Not serious	Not serious	Strong association	139/200 (69.5%)	95/176 (54.0%)	OR 2.08 (1.35-3.22)	169 more/1.000 (73-251; more-more)	High	Critical
Neurological adverse events (assessed with: Odds Ratio)												
2	RCT	Serious[a]	Serious[c]	Not serious	Not serious	None	18/307 (5.9%)	9/233 (3.9%)	OR 1.26 (0.55-2.89)	10 more/1.000 (17-65; fewer-more)	Low	Important
Infection-related adverse events (assessed with: Odds Ratio)												
2	RCT	Serious[a]	Not serious	Not serious	Not serious	None	78/307 (25.4%)	55/233 (23.6%)	OR 0.87 (0.56-1.36)	24 fewer/1.000 (89-60; fewer-more)	Moderate	Important
Gastrointestinal adverse events (assessed with: Odds Ratio)												
2	RCT	Serious[a]	Not serious	Not serious	Not serious	None	19/307 (6.2%)	9/233 (3.9%)	OR 1.36 (0.37-5.06)	13 more/1.000 (24-130; fewer-more)	Moderate	Important

a. The studies included in the meta-analysis presented a serious overall risk of bias, primarily due domain 5 of the RoB 2 tool, as outcomes were not pre-specified in study protocols.
b. There was no standardized time point defined for evaluating treatment response.
c. The definition of the outcome varied among the included studies.
